# Progression of Mental Health Services during the COVID-19 Outbreak in China

**DOI:** 10.7150/ijbs.45120

**Published:** 2020-03-15

**Authors:** Wen Li, Yuan Yang, Zi-Han Liu, Yan-Jie Zhao, Qinge Zhang, Ling Zhang, Teris Cheung, Yu-Tao Xiang

**Affiliations:** 1 Unit of Psychiatry, Institute of Translational Medicine, Faculty of Health Sciences, University of Macau, Macao SAR, China;; 2 Center for Cognition and Brain Sciences, University of Macau, Macao SAR, China;; 3 Department of Psychiatry, Southern Medical University Nanfang Hospital & Guangdong-Hong Kong-Macao Greater Bay Area Center for Brian Science and Brain-Inspired Intelligence, Guangdong, China;; 4 The National Clinical Research Center for Mental Disorders & Beijing Key Laboratory of Mental Disorders, Beijing Anding Hospital & the Advanced Innovation Center for Human Brain Protection, Capital Medical University, Beijing, China;; 5 School of Nursing, Hong Kong Polytechnic University, Hong Kong SAR, China

## Abstract

The novel coronavirus disease (COVID-19) has been rapidly transmitted in China, Macau, Hong Kong, and other Asian and European counterparts. This COVID-19 epidemic has aroused increasing attention nationwide. Patients, health professionals, and the general public are under insurmountable psychological pressure which may lead to various psychological problems, such as anxiety, fear, depression, and insomnia. Psychological crisis intervention plays a pivotal role in the overall deployment of the disease control. The National Health Commission of China has summoned a call for emergency psychological crisis intervention and thus, various mental health associations and organizations have established expert teams to compile guidelines and public health educational articles/videos for mental health professionals and the general public alongside with online mental health services. In addition, mental health professionals and expert groups are stationed in designated isolation hospitals to provide on-site services. Experts have reached a consensus on the admission of patients with severe mental illness during the COVID-19 outbreak in mental health institutions. Nevertheless, the rapid transmission of the COVID-19 has emerged to mount a serious challenge to the mental health service in China.

## The COVID-19 outbreak

The COVID-19 outbreak has been rapidly transmitted in late January 2020 and aroused enormous attention globally. The novel coronavirus disease (COVID-19) is formerly known as the '2019 Novel Coronavirus (2019-nCoV) Pneumonia', which was originated from a wet market in Wuhan, Hubei province, China in early December 2019 1. On the 7^th^ January, 2020, the Chinese Center for Disease Control and Prevention (CCDC) identified and isolated this novel coronavirus named severe acute respiratory syndrome coronavirus 2 (SARS-CoV-2). The SARS- CoV-2 can be transmitted between persons via close contacts 2 leading to COVID-19. Infected patients may develop severe and even fatal respiratory diseases (e.g., acute respiratory distress syndrome (ARDS) and acute respiratory failure) ending up in intensive care 2, 3. Due to potentially serious health outcomes brought by COVID-19, from 23^rd^ January, 2020, Wuhan and other regions in China have adopted strict quarantine measures to prevent and control the disease epidemic 4, 5. From 27^th^ January, 2020 onwards, all provinces, autonomous regions and municipalities in China have initiated an emergency response to major public health emergencies 4. On the 30^th^ January, 2020, the World Health Organization (WHO) declared the COVID-19 outbreak as a global health emergency 6. As of 18th February, 2020, the COVID-19 has caused 2,004 deaths out of the 74,185 confirmed cases 7. Worryingly, on 17^th^ February, 2020 the China CDC Weekly 8 reported that a total of 3,019 Chinese health workers have been infected with the SARS-CoV-2, of which, six of them died 9, 10 including Dr. Wen-Liang Li, a 'whistleblower' who first declared the possible emergence of severe acute respiratory syndrome pneumonia in China, and Dr. Zhi-Ming Liu, the President of Wuhan Wuchang Hospital in Hubei province.

## Mental health issues during the COVID-19 outbreak

Apart from physical suffering, it is not uncommon for confirmed or suspected cases of COVID-19 to suffer from great psychological pressure and other health-related problems. Health professionals are of no exception as they have the duty of care to the infected patients, close contacts with patients' families/ relatives, and sometimes, facing the public enquiry 11. Confirmed and suspected cases of the COVID-19 may experience fear of severe disease consequences and the contagion 11. Consequently, they may experience loneliness, denial, anxiety, depression, insomnia, and despair, which may lower treatment adherence. Some of these cases may even have increased risk of aggression and suicide. Suspected isolated cases may suffer from anxiety due to uncertainty about their health status and develop obsessive-compulsive symptoms, such as repeated temperature check and sterilization. Further, strict quarantine and mandatory contact tracing policy by health authorities could cause societal rejection, financial loss, discrimination, and stigmatization 12, 13. The limited knowledge of the COVID-19 and the overwhelming news may lead to anxiety and fear in the public 12, 14. The public at large may also experience boredom, disappointment, and irritability under the isolation measures 13.

Frontline health professionals, especially those in Wuhan, have close contact with infected patients. Excessive workload, isolation, and discrimination are frequently reported 15 and thus, they are highly vulnerable to experiencing physical exhaustion, fear, emotion disturbance, and sleep problems 15. A recent study involving 1,563 health professionals found that more than half (50.7%) of the participants reported depressive symptoms, 44.7% anxiety, and 36.1% sleep disturbance 16.

Patients with severe mental illness (SMI) are inevitably affected by the COVID-19 outbreak. In early February, 2020, over 40 inpatients in Wuhan Mental Health Center were diagnosed with the COVID-19 17. As of 18^th^ February, 2020, a total of 323 patients with SMI have been infected 17. In-patients, especially those requiring long-term hospitalization in closed wards, may experience fear of high risk of cluster contagion. Due to traffic restrictions and isolation measures, outpatients with SMI are facing difficulties to receive maintenance treatment, and may thus end up with mental relapse and uncontrollable behaviors (e.g., hyperactivity, agitation, and self- harm) 18. Patients with chronic diseases (e.g., chronic renal failure, diabetes mellitus, and cardio- cerebrovascular diseases), however, also need medical follow-up in hospitals regularly. These patients may also have difficulties to obtain maintenance treatment. As such, these preventive measures may lead to diminished physical wellbeing which exacerbates increased risk of negative feelings and suicidality. Families of deceased patients may suffer from bereavement and grief. The rapid transmission of the SARS-CoV-2 will increase the likelihood of mental distress and psychiatric morbidities in different sub-populations, not simply attributed to persistent quarantine and massive negative news portrayal, but also influenced by the growing number of confirmed and suspected cases, and death in China and the globe on a daily basis.

## Instructions and guidelines for mental health services during the COVID-19 outbreak

In order to reduce the risk of negative psychological outcomes caused by the COVID-19 outbreak and promote social stability, the National Health Commission of China (NHC) has integrated psychological crisis intervention into the general deployment of disease prevention. On the 27^th^ January, 2020, the central health authority issued the 'Principles for Emergency Psychological Crisis Intervention for COVID-19 Pneumonia Epidemic' 19. The Principle addressed that the guidelines should be implemented under the guidance of trained mental health professionals. Expert teams at province, autonomous region, and municipality level should take up the lead and responsibility on psychological crisis intervention and related activities. The mental health-related national associations and academic societies are required to adopt emergency psychological crisis interventions, psychological counseling and establish psychological assistance expert groups to provide professional guidance and coordinate with health authorities.

Following the aforementioned Principle, mental health associations and academic societies have organized expert teams, published guidelines and instructions for mental health services, such as the 'Psychosomatic Health Service Intervention Plan for the Prevention and Control of New Coronavirus Infection and Pneumonia (draft)' 20, and the 'Manual of National Mental Health of the New Coronavirus Pneumonia outbreak' 21. Online educational articles/videos are also released to the public, e.g., the article entitled 'Public Psychological Self-help Guideline for Pneumonitis with New Coronavirus Infection' 22, the series of 'New Coronavirus Science Popularization Knowledge' 23, the 'Online Lessons of Psychological Intervention during the New Coronavirus Pneumonia Outbreak' 24, and videos on 'Psychological Responses in the Fight Against New Crown Pneumonia' 25. The NHC also released the 'Psychological Adjustment Guidelines for Coping with the New Coronavirus Pneumonia' for specific populations, including older adults, children and adolescents, pregnant women and health professionals 26 (Table 1). In addition, mental health professionals in Wuhan have established expert consensus on psychological crisis intervention and uploaded videos of mental health education for the general public through WeChat and other internet platforms at the early stage of the outbreak 27, 28.

According to the Principle, psychological crisis interventions include three key points: 1) understanding the mental health status in different populations influenced by the COVID-19 outbreak; 2) identifying people who are at high risk of suicide and aggression, and 3) providing appropriate psychological interventions for those in need. Target populations are categorized in four levels: Level 1 population includes those who are most vulnerable to mental health problems, such as hospitalized patients with confirmed infection or severe physical condition, frontline health professionals and administrative staff. Level 2 population includes isolated patients with atypical infection symptoms (e.g., close contacts and patients with suspected infection) and patients at fever clinics. Level 3 population includes those individuals with close contacts in level 1 and 2, i.e., family members, colleagues, friends, and rear rescuers (organizational managers and volunteers) who participated in the COVID-epidemic response. Level 4 population includes people who are affected by the epidemic prevention and control measures, susceptible people, and the public.

## Clinical services for patients with severe mental illnesses during the COVID-19 outbreak

As for patients with SMI, the authorities released the 'Notice on Strengthening the Treatment and Management of Patients with Severe Mental Disorders during the Outbreak of the New Coronary Pneumonia' on the 17^th^ February, 2020 29. This notice suggested that: 1) mental health institutions should address prevention of nosocomial infections; 2) relevant departments should provide timely treatment and care for SMI patients infected with COVID-19, and 3) township/subdistrict-level integrated mental health management unit should provide management, treatment and community care for SMI patients at home.

Soon after the release of this notice, the Chinese Society of Psychiatry published the 'Expert Consensus on Managing Pathway and Coping Strategies for Patients with Mental Disorders during Prevention and Control of Infectious Disease Outbreak (Novel Coronavirus Pneumonia)' 18. This expert consensus addressed that mental health institutions should strictly implement the 'Technical Guidelines for the Prevention and Control of Novel Coronavirus Infection in Medical Institutions (1st edition)' 30, the 'Diagnosis and Treatment Protocol for Novel Coronavirus Pneumonia (revised pilot version 5)' 31, and related infectious disease protection requirements. This expert consensus also provided suggestions for hospital management during the COVID-19 outbreak for both psychiatric hospitals and psychiatric units in general hospitals. For example, psychiatric hospitals should reduce outpatient visits, tighten admission criteria, and shorten the length of hospitalization. For newly admitted psychiatric patients, isolation wards should be set up and visiting should be suspended to minimize the potential risk of nosocomial infection.

## Specific measures in mental health services during the COVID-19 outbreak

Considering the traffic limits and quarantine measures in many areas, online mental health services have been widely adopted, such as hotlines and mobile application platforms. For example, the Psychological Rescue Branch of Chinese Association for Disaster and Emergency Rescue Medicine developed the 'National Crisis Intervention Platform for New Coronavirus Pneumonia' at the Second Xiangya Hospital of Central South University on the 31^th^ January, 2020 32. The 'Supporting System of Strengthening Lung and Mental Health' has also been established by the Renmin Hospital of Wuhan University. This system provides online consulting services and assigned crisis response teams to provide mental health education for both patients and frontline medical staff 33. The University of Macau established a team of psychological professionals who assisted the Social Work Bureau to provide psychological counseling via hotline, WeChat, and video for isolated persons 34. In addition, psychologists and psychiatrists provide individual counseling on ePlatforms (e.g., Alihealth and HaoDaiFu) and adopt strategies to relieve stress via social media (e.g., Weibo and WeChat) 14, 35. In order to effectively supervise online psychological crisis intervention, the NHC released the 'Notices on Establishing Psychological Assistance Hotlines for COVID-19 Outbreak' on the 2^th^ February, 2020 36, and the Bureau of Disease Prevention and Control released the 'Guidelines for Psychological Assistance Hotlines during the New Coronavirus Pneumonia Outbreak' on the 7^th^ February, 2020 37. The guidelines addressed that the hotline services should be set up by psychiatric institutions, students' mental health education and counseling centers at universities, mental health associations and academic societies. The qualifications and responsibilities of the consultants and the supervisors in the hotline services have also been stipulated. Moreover, the Chinese Psychological Society published the 'Work Guideline for Psychological Assistance Hotline during the COVID-19 Outbreak' 38 and the 'Guidelines for Internet Psychological Counseling during Special Epidemic Period' 39 to provide professional assistance for online psychological services (Table 1). Apart from online mental health services, hundreds of psychologists and psychiatrists, alongside with the emergency rescue team went to Wuhan, China 40, 41 to provide on-site psychological counseling and professional guidance.

## Conclusions

The outbreak of the COVID-19 has caused tremendous psychological problems in different subpopulations. Despite the Chinese authorities have announced relevant policies and actuating principles in a strategic manner, the COVID-19 outbreak has posted an emerging serious challenge for the mental health services in China. There are some limitations that need to be addressed. First, several online mental health services have been constructed across different areas, however, unified national management and coordination policies are still inadequate, which could result in uneven distribution and wastage of medical resources, let alone evaluating the efficacy of these services. Second, online mental health services are the predominant assistance measure and therefore, some people (e.g., older adults) who may have limited access to smartphones and the broadband internet, may benefit less from the services 42. Third, frontline health professionals may have limited time and energy to access to these services due to heavy workload. Fourth, based on experiences of SARS outbreak, some patients and health professionals would be traumatized by the COVID-2019 outbreak and still suffer from persistent psychiatric symptoms even after the outbreak 43. Thus, the COVID-19 epidemic has become an extra socio-economic burden on existing mental health service provision in China. Finally, Chinese researchers published papers on English-language international journals regarding the latest development of online mental health services. Local frontline health professionals and policymakers may not be able to benefit from these findings due to the language barrier 44.

## Figures and Tables

**Table 1:** Summary of guidelines and instructions for mental health services during the COVID-19 outbreak in China between 26^th^ Jan and 20^th^ Feb, 2020

Date of publication	Organizations	Gidelines and instructions	References
26^th^ Jan	Medical College of Tongji University Dongfang Hospital; China International Emergency Medical Team (Shanghai)	'Mental Health and Humane Care Issues Related to the COVID-19 Infection' was published	35
27^th^ Jan	National Health Commission of China Bureau of Disease Prevention and Control	'Principles for Emergency Psychological Crisis Intervention for COVID-19 Pneumonia Epidemic' was published	19
28^th^ Jan	National Health Commission of China Bureau of Disease Prevention and Control	'Interpretation of Principles of Emergency Psychological Crisis Intervention for the COVID-19 Epidemic' was published	45
		'A Picture to Read: Principles of Emergency Psychological Crisis Intervention for the COVID-19 Epidemic' was published	46
31^st^ Jan	Source: Joint Psychological Mission of the Chinese Psychiatrist Association and the Chinese Society of Psychiatry Review: Chinese Association for Mental Health Crisis Intervention Committee	'[New Coronavirus Science Popularization Knowledge] (54) How to help frontline medical staff to relieve their psychological pressure' was published	23
31^st^ Jan	Psychological Rescue Branch of Chinese Association for disaster & Emergency Rescue Medicine	'National Crisis Intervention Platform for New Coronavirus Pneumonia' was developed	32
31^st^ Jan	Chinese Psychological Society	'Work Guideline for the Psychological Assistance Hotline during the COVID-19 Outbreak (draft)' was published	38
1^st^ Feb	Source: Joint Psychological Mission of Chinese Psychiatrist Association and Chinese Society of Psychiatry Review: Chinese Association for Mental Health Crisis Intervention Committee	'[New Coronavirus Science Popularization Knowledge] (66) How should the public face the psychological panic caused by the outbreak?' was published	23
1^st^ Feb	Chinese Society of Psychiatry	'Psychological Adjustment for Frontline Medical Workers: Suggestions from the Chinese Society of Psychiatry' was published	47
2^nd^ Feb	National Health Commission of China Bureau of Disease Prevention and Control	'Notices on Establishing Psychological Assistance Hotlines for COVID-19 Outbreak' was published	36
		'Interpretation of the Notice on Establishing Psychological Assistance Hotlines for the COVID-19 Outbreak' was published	36
2^nd^ Feb	Source: Joint Psychological Mission of Chinese Psychiatrist Association and Chinese Society of Psychiatry Review: Chinese Association for Mental Health Crisis Intervention Committee	'[New Coronavirus Science Popularization Knowledge] (71) How to use the Internet to complete self-psychological intervention?' was published	48
3^rd^ Feb	Chinese Society for Psychosomatic	'Psychosomatic Health Service Intervention Plan for the Prevention and Control of New Coronavirus Infection and Pneumonia (draft)' was published	20
3^rd^ Feb	Chinese Association of Mental Health	'Public Psychological Self-Help Guideline for Pneumonitis with New Coronavirus Infection' was published	22
3^rd^ Feb	Chinese Psychological Society	'Guidelines for Internet Psychological Counseling during Special Epidemic Period (1)' was published	39
7^th^ Feb	National Health Commission of China Bureau of Disease Prevention and Control	'Psychological Adjustment Guidelines for Coping with the New Coronavirus Pneumonia' was published	26
7^th^ Feb	Bureau of Disease Prevention and Control State Council Joint Prevention and Control Team to Response to New Coronavirus Pneumonia	'Guidelines for Psychological Assistance Hotlines during the New Coronavirus Pneumonia Outbreak' was published	37
8^th^ Feb	Chinese Psychological Society	Videos of 'Psychological Responses in the Fight against New Crown Pneumonia' was released	25
8^th^ Feb	Chinese Association for Physiological Sciences	'Leverage the Advantages to Fight against the Epidemic - Reports of the Chinese Association of Physiological Sciences' was published	49
8th-9th Feb	Chinese Society of Psychiatry Central Radio and television Station	Video of 'Online Lessons of Psychological Intervention during the New Coronavirus Pneumonia Outbreak' was uploaded	24
10th Feb	Chinese Psychological Society	'Psychological Construction Manual for Enterprises, Organizations and Institutions Dealing with the Outbreak was published	50
12th Feb	Chinese Psychological Society Chinese Association for Mental Health Chinese Association of Social Psychology	'Guidelines for Online Psychological Assistance Services during the Prevention and Control of Novel Coronavirus Pneumonia was published	51
14^th^ Feb	Editor in chief: Lu Lin, Wang Gao-Hua Organization: China Health Education Center, National Health Commission of China, Bureau of Disease Prevention and Control Chinese Physicians' Association.	'Manual of National Mental Health of the New Coronavirus Pneumonia Outbreak' was published	21
17^th^ Feb	Integrated group of joint prevention and control mechanism for the outbreak of new coronavirus pneumonia of the state council of China	'Notice on Strengthening the Treatment and Management of Patients with Severe Mental Disorders during the Outbreak of the New Coronary Pneumonia' was released	29
18^th^ Feb	Chinese Society of Psychiatry	'Expert Consensus on Managing Pathway and Coping Strategies for Patients with Mental Disorders during Prevention and Control of Infectious Disease Outbreak (Novel Coronavirus Pneumonia)' was published	18
18^th^ Feb	National Health Commission of China	Interpretation of 'Notice on Strengthening the Treatment and Management of Patients with Severe Mental Disorders during the Outbreak of the New Coronary Pneumonia' was released	52
18^th^ Feb	Bureau of Disease Prevention and Control, National Health Commission of China Peking University Six Hospital National Mental Health Program Office Chinese Center for Disease Control and Prevention	[New Coronavirus Science Popularization Knowledge] (184) How do pregnant women adjust their psychological status?	53
		[New Coronavirus Science Popularization Knowledge] (185) How do relatives of those who died from the outbreak adjust their psychological status?	54
20^th^ Feb	Bureau of Disease Prevention and Control, National Health Commission of China Peking University Six Hospital National Mental Health Program Office Chinese Center for Disease Control and Prevention	[New Coronavirus Science Popularization Knowledge] (198) How do employees who have returned to work release their anxiety?	55
